# Effects of *Bacillus licheniformis* on the water quality, growth performance and bacterial community in *Penaeus vannamei* aquaculture system

**DOI:** 10.3389/fmicb.2025.1595680

**Published:** 2025-06-02

**Authors:** Yi Zheng, Yingzi Wu, Shuling Tang, Junpeng Li, Lingyu Fan, Wenjin He

**Affiliations:** ^1^College of Life Sciences, Fujian Normal University, Fuzhou, Fujian, China; ^2^National Joint Engineering Research Center of Industrial Microbiology and Fermentation Technology, College of Life Sciences, Fujian Normal University, Fuzhou, Fujian, China

**Keywords:** *Penaeus vannamei* aquaculture, *Bacillus licheniformis*, high-throughput sequencing, bacterial community analysis, water quality, growth performance

## Abstract

**Introduction:**

This study investigated the effects of *Bacillus licheniformis* on the water quality, growth performance and bacterial community in *Penaeus vannamei* aquaculture system. The objective was to elucidate the impact of *B. licheniformis* on *P. vannamei* aquaculture from a microbial ecological perspective. This research provided valuable theoretical support for the practical application of *B. licheniformis* in improving aquaculture practices for *P. vannamei*.

**Methods:**

The design of the aquaculture experiment comprised two groups: a control group (CK) fed a basal diet and a treatment group (PB) was fed the same diet with addition of *B. licheniformis* (5 × 10^4^ CFU/mL) into the water every 5 days. These groups were systematically evaluated through comprehensive water quality analyses, including pH, ammonia nitrogen, nitrite nitrogen, and Vibrio counts, as well as growth performance assessments such as length, weight, survival rate, yield, and feed conversion ratio (FCR). Additionally, high-throughput sequencing technology was employed to analyze changes in bacterial community structures in both the aquaculture water and the shrimp intestines.

**Results:**

The results demonstrated that *B. licheniformis* significantly improved water quality, promoted shrimp growth, and altered the bacterial community structure: (1) *B. licheniformis* significantly reduced the concentrations of ammonia nitrogen, nitrite nitrogen, and pathogenic *Vibrio* counts in the later stages of cultivation (*P* < 0.05), while significantly promoting shrimp growth; (2) The addition of *B. licheniformis* increased the diversity and richness of bacteria both in the water and shrimp intestinal tracts, leading to significant changes in bacterial community structure. It also enhanced beneficial bacterial genera such as *Gemmobacter, Paracoccus*, and *Bacillus* in the water, while concurrently reducing the potential pathogenic *Flavobacterium* in the shrimp intestinal tract; (3) The dominant bacterial populations were significantly affected in both water and shrimp intestinal samples. In water, *Aurantimicrobium* was the predominant genus in both groups, with the PB group showing a notably lower relative abundance. In the shrimp intestines, the CK group was dominated by *Gemmobacter* and *Fluviicola*, while *Aurantimicrobium* prevailed in the PB group. In conclusion, the study revealed the potential of *B. licheniformis* in shrimp aquaculture by improving water quality, promoting shrimp growth, and modulating bacterial community structure.

**Discussion:**

This study demonstrated that *B. licheniformis* significantly improved water quality and shrimp growth performance in *P. vannamei* aquaculture. It effectively reduced pH, ammonia and nitrite levels, while also decreasing *Vibrio* counts, which are critical for disease control. *B. licheniformis* enhanced microbial diversity in both aquaculture water and shrimp intestines, promoting the abundance of beneficial genera, while reducing potential pathogens. The alteration in bacterial community structure suggests that *B. licheniformis* plays a pivotal role in maintaining a healthy microbial ecosystem, enhancing shrimp health and growth, and providing an environmentally sustainable alternative to antibiotics in aquaculture practices. These findings underscore the potential of *B. licheniformis* for improving *P. vannamei* aquaculture systems.

## 1 Introduction

*Penaeus vannamei*, native to the Pacific coast of South America (Liao and Chien, 2011), is one of the most economically significant cultured shrimp species globally (FAO, 2024). Recent advancements in living standards and increasing demands for diverse nutritional sources have driven rapid development in shrimp aquaculture (Cabello et al., 2016). However, the high-density intensive shrimp farming model, while economically beneficial, has precipitated several challenges, including the deterioration of water quality (Duan et al., 2018) and the frequent outbreak of diseases (Le et al., 2024). In recent years, bacterial diseases such as acute hepatopancreatic necrosis disease (AHPND), caused by *Vibrio parahaemolyticus*, have severely disrupted the shrimp industry. In regions affected by AHPND, particularly in Asia and South America, shrimp production has declined by ~ 60%. This disease has resulted in an annual global loss of $1 billion to the shrimp farming industry (Kumar et al., 2018). Consequently, enhancing water quality and bolstering shrimp resistance to pathogenic bacteria are critical for sustaining healthy shrimp aquaculture.

In the aquaculture industry, chemicals such as antibiotics and disinfectants were once widely used in intensive shrimp aquaculture as the preferred means to control the prevalence of diseases and maintain high production (Li et al., 2021). However, the increased use of antibiotics has revealed significant drawbacks: on the one hand, it can lead to the development of antibiotic resistance in pathogenic microorganisms (Cabello, 2006), causing drug accumulation that paralyzes the entire food chain, damages the aquatic environment, and leads to unsustainable development. On the other hand, it can disrupt the balance of the intestinal microbiota in shrimp, negatively affecting the host's immune response and growth performance (Chen et al., 2023). Therefore, there is an urgent need for modern intensive aquaculture to explore better disease prevention and control methods, and to seek safer and more environmentally friendly alternatives to antibiotics, to enhance aquaculture efficiency and safeguard human health.

The use of probiotics has become an alternative approach to antibiotics in aquaculture. Probiotics are bacterial-based products that are eco-friendly, non-polluting, and do not induce antimicrobial resistance. These characteristics make them widely used in shrimp farming as a new tool for promoting health and preventing diseases (Kewcharoen and Srisapoome, 2019; Ringø, 2020). Additionally, the application of non-pathogenic bacteria can enhance dietary safety and environmental performance by modulating host-associated microbiota, which helps control allergic reactions and strengthens immune system function (Van Doan et al., 2020; Llewellyn and Foey, 2017).

Among the various probiotics utilized in aquaculture, *Bacillus licheniformis* is widely acknowledged for its safety and efficacy in animal feed. This bacterium, a prominent member of the *Bacillus* genus, is distinguished by its robust spore-forming capability, which ensures its survival in both harsh environmental conditions and the gastrointestinal tract of aquatic organisms (Fan et al., 2021). *B. licheniformis* has been extensively investigated for its significant role in ecological regulation and its application in aquaculture (Abarike et al., 2018; Chen et al., 2020; Ma et al., 2022; Monier et al., 2023; Vega-Carranza et al., 2024). Currently, related studies primarily focus on the effects of improving aquaculture water quality and increasing production yields. However, in-depth studies investigating the mechanisms of probiotics within *P. vannamei* aquaculture systems from a microbial ecology perspective remain relatively limited.

In this study, *B. licheniformis* strain FS051 was added to the aquaculture water of *P. vannamei*. The effects of *B. licheniformis* on water quality and shrimp growth performance were comprehensively evaluated. High-throughput sequencing technology was employed to analyze changes in bacterial community structures in both the aquaculture water and the shrimp intestines. The aim was to elucidate the impact of *B. licheniformis* on *P. vannamei* aquaculture from a microbial ecological standpoint. This research provided theoretical support for the application of *B. licheniformis* in *P. vannamei* aquaculture.

## 2 Materials and methods

### 2.1 Experimental materials

#### 2.1.1 Bacterial strain

*Bacillus licheniformis* strain FS051, was previously isolated from the intestines of healthy *Penaeus vannamei* in Pingtan, Fujian Province, China, by the laboratory (College of Life Sciences, Fujian Normal University). This strain exhibited both nitrite-degrading ability and antibacterial activity against *Micrococcus luteus*. The purity of the strain was confirmed through the observation of its morphological and biochemical characteristics, as well as molecular identification of the species.

#### 2.1.2 Shrimp species

The juvenile *Penaeus vannamei* with an initial weight of 0.80 to 0.90 g and a body length of 3.0 to 4.0 cm, were obtained from an aquaculture farm in Pingtan, Fujian Province, China.

### 2.2 Experimental set-up and rearing condition

The juvenile *P. vannamei* were randomly distributed into two groups (in triplicate) in culture tanks (56 cm × 41 cm × 32.5 cm, water depth: 30 cm). Each tank was equipped with an oxygenation device and fitted with a nylon cloth filter of 500 mesh size. The stocking density was set at 30 shrimp per tank. The animals were fed 3–5% of their live body weight for three times (morning, noon, and evening) a day with commercialized feed, primarily composed of fish meal, yeast, peanuts, soybean meal, and others. The commercial feed containing crude protein 43%, crude fat 6%, ash 16%, and water 12%. The experiment lasted for 30 days.

The aquaculture water was prepared by diluting seawater with tap water at a ratio of 11:1.4, maintaining a salinity of ~ 13‰. No water exchange occurred throughout the cultivation process.

Each treatment group were as follows: the CK group did not add any microbial preparations and was managed routinely, the PB group indicated that *B. licheniformis* was added to the water every 5 days during the aquaculture process at a dosage of 5 × 10^4^ CFU/mL.

### 2.3 Water quality measurement methods

(1) Determination of water pH : the water pH was directly determined by a pH meter (PHS-3C, Leici, Shanghai, China).
(2) Determination of ammonia nitrogen in water: samples were taken from each tank at a depth of 10 cm, and after centrifugation at 8,000 r/min for 10 min, the concentration of ammonia in the water samples was determined using the Nessler's reagent colorimetric method (Wu and Cao, 2013), with an ultraviolet spectrophotometer (UV-1800, Shimadzu, Suzhou, China).
(3) Determination of nitrite nitrogen in water: the concentration of nitrite nitrogen was determined by the diazotization-azo coupling colorimetric method.
(4) Determination of *Vibrio* counts in water: after diluting the culture water to 10^−1^ and 10^−2^ gradient, 0.1 mL of the original solution and the 10^−1^ and 10^−2^ gradient solutions were plated onto TCBS solid culture medium. Colony counts between 30 and 300 were selected and expressed using the CFU counting method.

### 2.4 Measurement of shrimp-related indicators

(1) Measurement of shrimp length and weight: shrimp length was measured with a straightedge with an accuracy of 0.01 cm, and weight was measured with an electronic balance with an accuracy of 0.01 g.
(2) Measurement of shrimp yield, feed conversion ratio (FCR) and survival rate: shrimp yield was expressed as the total fresh weight of all shrimp at the end of the experiment. The shrimp survival rate was determined as the ratio of the final number of shrimp to the initial number of shrimp. The FCR was calculated using the formula:

(1) $$\begin{array}{l} \text{FCR}=\frac{\text{total feed intake }(\text{g})}{\text{total wet weight gain }(\text{g})} \end{array}$$

### 2.5 Samples collection

At the end of the trial, the shrimp and the environment were sampled. Six shrimp were randomly taken from each tank. The shrimp were placed in a −20°C refrigerator to freeze for 20 min to death and then transferred to a 4°C refrigerator for cold storage. Then they were soaked in 75% alcohol for 30 s and rinsed with sterile water. In a clean bench, the shrimp intestinal tracts were extracted using sterile tweezers and packed into sterile centrifuge tubes, sealed, and stored at −80°C.

A sterile sampler was used to take a sample of 500 mL at a water depth of 10 cm in each culture tank, which was first filtered with a 500-mesh nylon cloth for primary filtration, and after the primary filtration, it was filtered with a 0.22 μm filter membrane for secondary filtration, and after filtration, the membrane was put into a 50 mL sterile centrifuge tube and stored at −80 °C.

Subsequently, the shrimp intestinal tract samples and the water samples were subjected to high-throughput sequencing.

### 2.6 DNA extraction and PCR amplification

After extracting the DNA of the samples by CTAB, the quality and purity of DNA were assessed through 1% agarose gel electrophoresis. The concentration of DNA was diluted to 1 ng·μL^−1^ to serve as a template for PCR amplification. The PCR reaction was performed using Phusion^®^ High-Fidelity PCR Master Mix with GC Buffer, along with high-performance, high-fidelity enzymes, and the prokaryotic universal primers 341F and 806R. The primers and sequences utilized for PCR amplification were as described below:

341F (5'-CCTAYGGGRBGCASCAG-3')

806R (5'-GGACTACNNGGGTATCTAAT-3')

The amplified products were analyzed using 1% agarose gel electrophoresis to determine the sizes of the amplified bands. Subsequently, the PCR products were purified using the Agencourt AMPure XP nucleic acid purification kit.

### 2.7 High-throughput sequencing and sequence data processing

The library was constructed using the Ion Plus Fragment Library Kit 48rxns from Thermo Fisher. After library construction, quantification was performed using Qubit, and libraries that passed quality control were sequenced on the Thermo Fisher Life Ion S5™ system.

The original sequences were analyzed using the QIIME software, with end-splicing accomplished by FLASH. Preliminary quality control was conducted through UPARSE, removing low-quality sequences, followed by clustering of all effective tags into OTUs and species annotation of representative OTU sequences. For the top 20 genera in abundance, heatmaps were generated using R language. The QIIME package was used to calculate alpha diversity for each sample, and principal component analysis (PCA) and other graphical representations were generated using the VennDiagram package.

### 2.8 Statistical analysis

The experimental data were presented as the mean ± standard error. The independent-samples *t*-test and one-way analysis of variance (ANOVA) were used to identify significant differences using SPSS software. A level of *P* < 0.05 was accepted as statistically significant. The data processing was conducted using Excel 2016. Graphs were generated using Origin 2018 software.

## 3 Results and analysis

### 3.1 Effects of *B. licheniformis* on the water quality

#### 3.1.1 Changes in pH in the culture water

**Figure 1:**
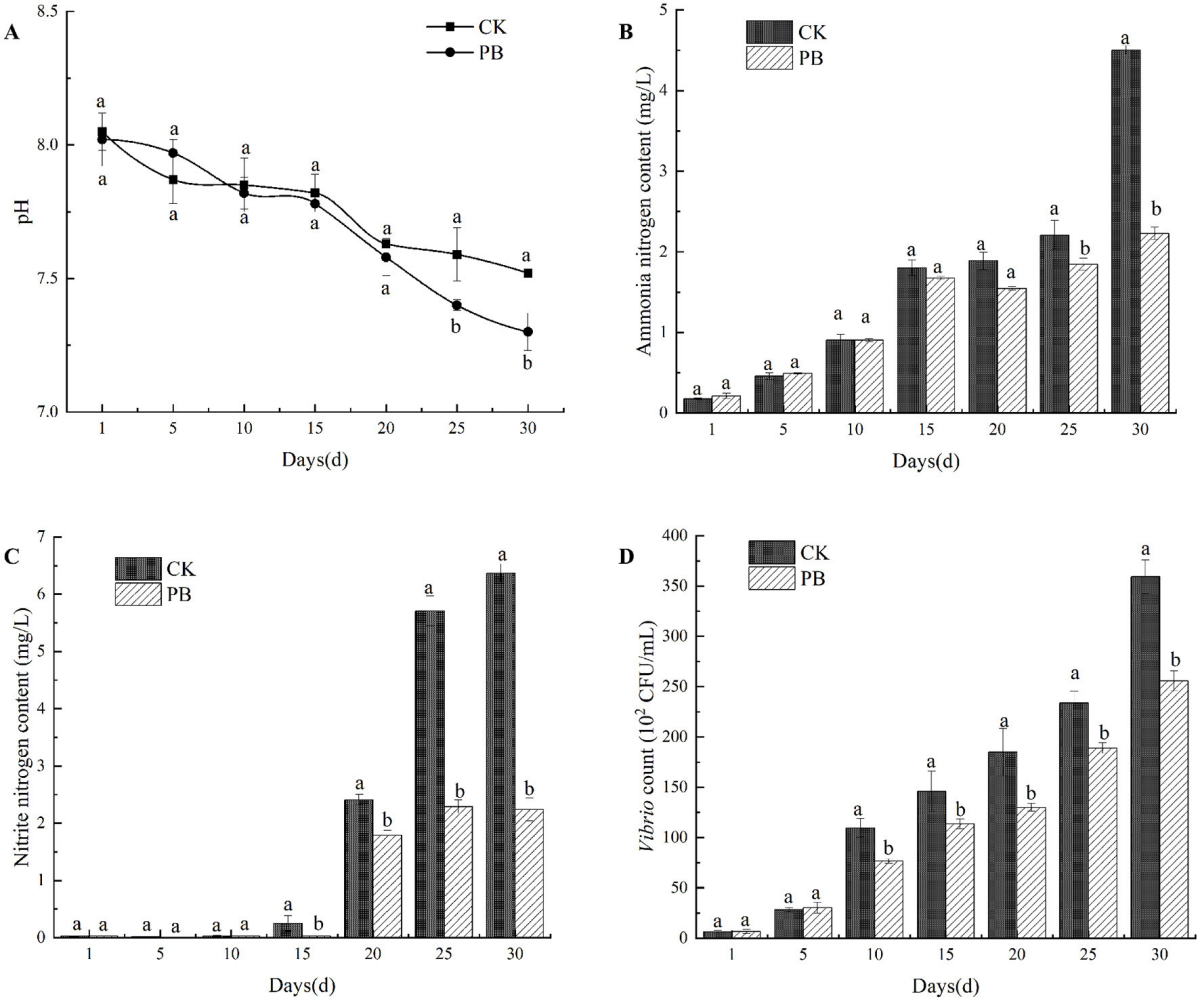
Effects of *Bacillus licheniformis* on the water quality. **(A)** pH variations in the culture water of CK and PB groups. **(B)** Changes of ammonia nitrogen content in different treatment groups. **(C)** Changes of nitrite nitrogen content in different treatment groups. **(D)** Changes of *Vibrio* count in different treatment groups. CK: no probiotic added; PB: *B. licheniformis* added to the water. Different letters within the same time point indicate statistically significant differences (*P* <0.05). Error bars represent standard deviations.

*P. vannamei* exhibits strong adaptability to its environment, with its optimal growth pH range is between 7.5 and 8.5 (Chen and Chen, 2003). During the culture process, the pH was measured every 5 days. With the extension of the culture time, the CK group and the PB group showed a decreasing trend in pH, and both of them had basically the same trend of change, with the pH maintained between 7.3 and 8.1 (Figure 1A).

The experimental results indicated that compared to the CK group, *B. licheniformis* lowered water pH for *P. vannamei*, but it remained within the optimal range for shrimp growth. There was no significant difference between the two groups in the early stages, while in the later stages, the pH in the PB group was significantly lower than that in the CK group (*P* < 0.05).

#### 3.1.2 Changes in ammonia and nitrite levels in the culture water

During the culture process, the concentrations of ammonia and nitrite in the water of each treatment group were measured every 5 days. In the first 10 days of aquaculture, the ammonia concentration maintained at low levels (Figure 1B), consistently below 1 mg/L, with no significant difference between the CK group and the PB group. However, from day 10 to day 20, the ammonia levels began to rise sharply, peaking at 4.50 mg/L on day 30. Notably, on the 25th day and the 30th day, the ammonia levels in the PB group were significantly lower than those in the CK group (*P* < 0.05), indicating that *B. licheniformis* significantly reduced the ammonia concentration in water in the later stages of culture.

The nitrite concentration maintained at low levels during the first 5 days of aquaculture (Figure 1C), not exceeding 0.5 mg/L. After the 15th day, the nitrite levels in both the CK group and the PB group began to increase, reaching their highest levels on day 30. The nitrite concentration in the PB group was significantly lower than that in the CK group after the 15th day (*P* < 0.05), indicating that *B. licheniformis* significantly reduced the nitrite concentration in the water during the later stages of aquaculture.

#### 3.1.3 Variations of *Vibrio* counts in the culture water

*Vibrio* is a bacterial pathogen in *P. vannamei* aquaculture that can cause red leg disease, with diseased shrimp displaying symptoms such as reddened appendages, slow movement, and anorexia (Sudheesh and Xu, 2001). *Vibrio* counts in the water were measured every 5 days (Figure 1D). The counts were determined by plating diluted water samples onto TCBS solid culture medium and counting the CFU. The data showed a gradual increase in *Vibrio* counts for both the CK and PB groups throughout the culture process. In the first 10 days, *Vibrio* counts remained below 100 × 10^2^ CFU/mL, with no significant difference between the CK group and PB group. After day 10, *Vibrio* counts increased, peaking on day 30 at 3.60 × 10^4^ CFU/mL for the CK group and 2.56 × 10^4^ CFU/mL for the PB group. From day 10 to 30, *Vibrio* counts in the PB group were significantly lower than that in the CK group (*P* < 0.05), demonstrating that *B. licheniformis* significantly reduced the number of *Vibrio* in the aquaculture water during the later stages of *P. vannamei* aquaculture.

### 3.2 Impacts of *B. licheniformis* on the growth of *P. vannamei*

The growth parameters of shrimp were measured (Table 1). The data clearly indicated that *B. licheniformis* significantly enhanced growth indicators (*P* < 0.05), including shrimp length, weight, survival rate, and overall yield compared to the CK group. FCR represents the feed required to cultivate 1 kg of shrimp, and serves as a crucial efficiency indicator. A lower FCR indicates better feed conversion. As shown in the table, the PB group's FCR was significantly lower than the CK group's. In summary, *B. licheniformis* promoted shrimp growth and improved feed conversion.

**Table 1:** Growth indices of *Penaeus vannamei* in different treatment groups.

**Group name**	**Shrimp length (cm)**	**Weight (g)**	**Survival rate (%)**	**FCR**	**Overall yield (g)**
CK	8.39 ± 0.16b	3.48 ± 0.07b	72.22% ± 5.09b	0.85 ± 0.09a	75.36 ± 6.05b
PB	9.44 ± 0.08a	4.74 ± 0.11a	91.11% ± 6.94a	0.49 ± 0.04b	129.70 ± 12.55a

CK: no probiotic added; PB: *B. licheniformis* added to the water. Different letters indicate statistically significant differences between different treatment groups (*P* < 0.05).

### 3.3 Influences of *B. licheniformis* on bacterial diversity in culture water and shrimp intestinal tract

#### 3.3.1 Bacterial diversity indices of the culture water and the shrimp intestinal tracts in different treatment groups

Alpha diversity analysis was conducted on bacterial communities in the culture water and the shrimp intestinal tracts using high-throughput sequencing technology. By comparing the diversity indices of different treatment groups (Table 2), the numbers of OTUs were 195, 230, 194, and 231, respectively. The library coverage was 100%, indicating a high detection rate and reliable reflection of actual bacterial communities in the aquaculture water and shrimp intestines. In the water samples, the W_PB group had higher Chao1, Shannon, and Simpson indices than the W_CK group. Similarly, the S_PB group had higher indices than the S_CK group in shrimp intestinal tract samples. Therefore, *B. licheniformis* enhanced bacterial community richness in both the water and shrimp intestinal tract samples.

**Table 2:** Diversity index of different treatment groups.

**Sample**	**Diversity analysis**				
	**Chao1**	**Shannon**	**Simpson**	**OTUs**	**Coverage**
W_CK	247.45	18.06	0.83	195	100%
W_PB	287.60	19.69	0.84	230	100%
S_CK	219.05	18.45	0.72	194	100%
S_PB	266.44	20.37	0.87	231	100%

W, water; S, shrimp. Here, W_CK and S_CK represent samples obtained from water and shrimp intestine samples from the untreated group, whereas W_PB and S_PB represent samples from the probiotic treated group.

#### 3.3.2 The OTU distribution among different treatment groups

**Figure 2:**
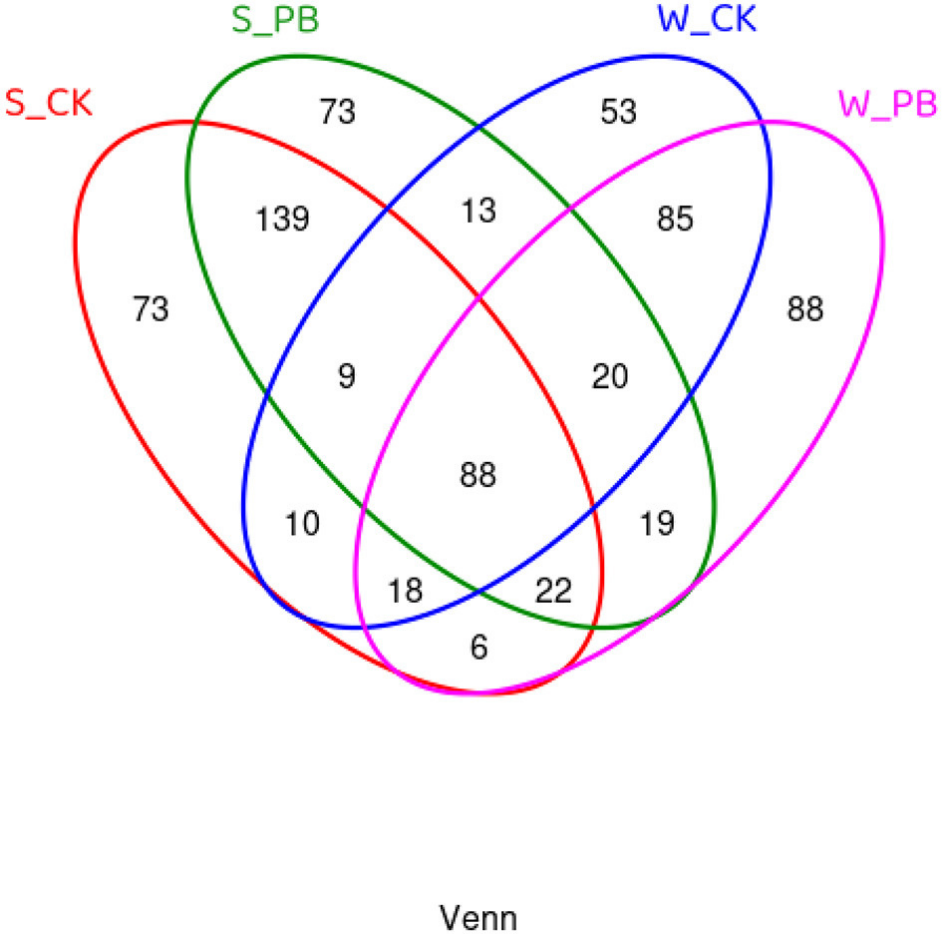
Venn diagram of OTU distribution, showing shared and unique OTU among different treatment groups. W, water; S, shrimp. Where W_CK; S_CK represents samples obtained from water and shrimp intestine samples obtained from untreated group and W_PB; S_PB probiotic treated group.

Using Venn diagrams to compare the shared and unique OTUs among different treatment groups (Figure 2), it was evident that in the water samples, the W_CK group and the W_PB group possessed 53 and 88 unique OTUs, respectively, with 85 OTUs shared between them. In the shrimp intestinal tract samples, both the S_CK and S_PB groups had 73 unique OTUs each, with 139 OTUs shared between them. These results indicated that *B. licheniformis* altered the microbial composition in the aquaculture water and the shrimp intestines.

### 3.4 Influences of *B. licheniformis* on the composition of bacterial community in the culture water and shrimp intestine

#### 3.4.1 Bacterial community composition at the phylum level in the aquaculture water

**Figure 3:**
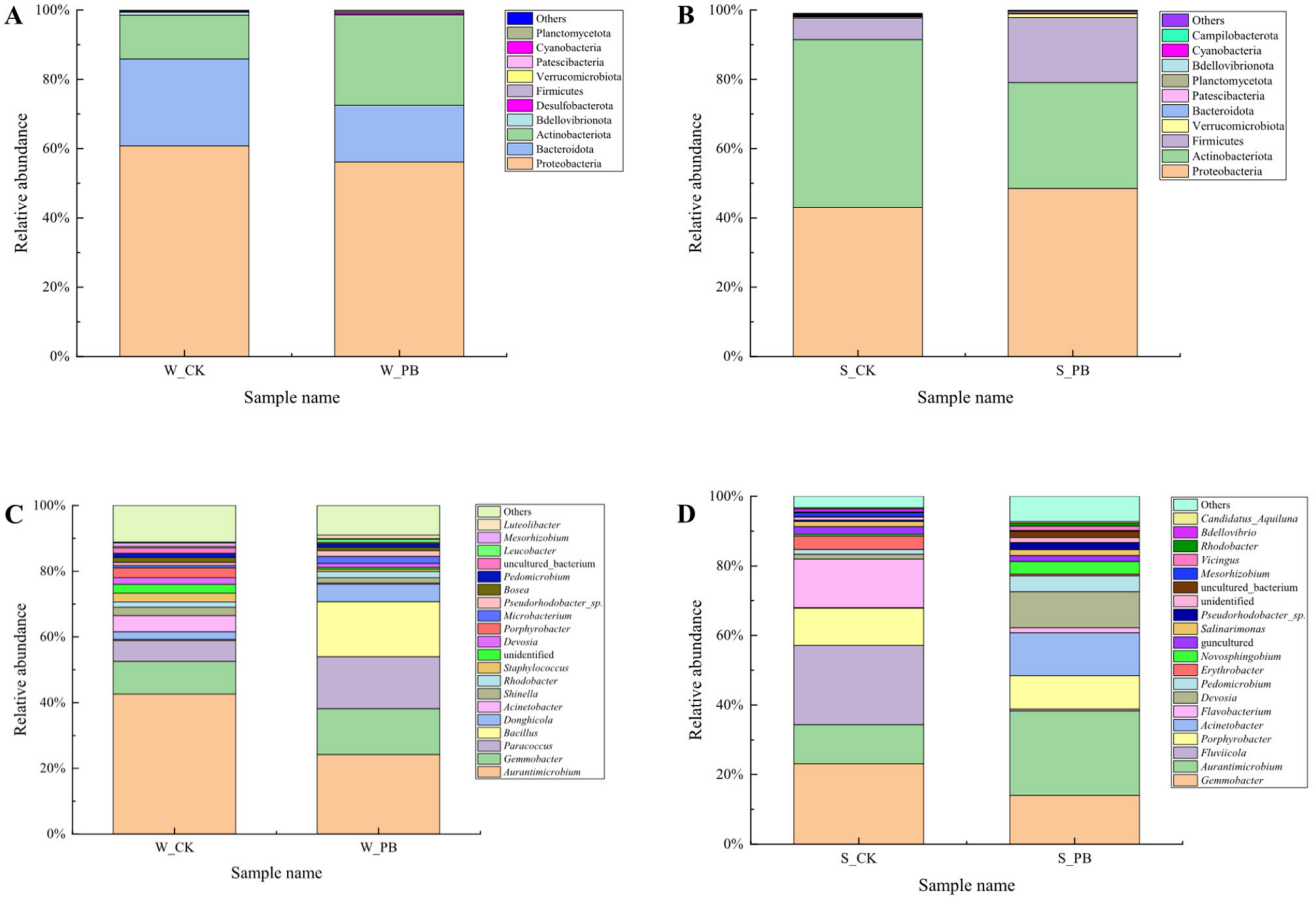
Bacterial community composition in different treatment groups. **(A)** Phylum-level composition in culture water, W_CK: the untreated group of water samples; W_PB: the probiotic treated group of water samples. **(B)** Phylum-level composition in shrimp intestinal tract, S_CK: the untreated group of shrimp intestinal tract samples; S_PB: the probiotic treated group of shrimp intestinal tract samples. **(C)** Genus-level composition in culture water, W_CK: the untreated group of water samples; W_PB: the probiotic treated group of water samples. **(D)** Genus-level composition in shrimp intestinal tract, S_CK: the untreated group of shrimp intestinal tract samples; S_PB: the probiotic treated group of shrimp intestinal tract samples.

Analyzing the bacterial community composition at the phylum level in the water samples (Figure 3A). The top 10 phyla accounted for 99.98% to 99.99% of the total relative abundance. These phyla included Proteobacteria, Bacteroidota, Actinobacteriota, Bdellovibrionota, Desulfobacterota, Firmicutes, Verrucomicrobiota, Patescibacteria, Cyanobacteria, and Planctomycetota. Of which Proteobacteria, Bacteroidota, Actinobacteriota, and Bdellovibrionota were the dominant phyla, accounting for 98.68% to 99.42% of the total. Compared to the W_CK group, the W_PB group exhibited a decrease in the relative abundance of Proteobacteria, Bacteroidota, and Bdellovibrionota, while Actinobacteriota increased. This indicated that the *B. licheniformis* altered the bacterial community structure in the aquaculture water.

#### 3.4.2 Bacterial community composition at the phylum level in shrimp intestinal tracts

Analyzing the bacterial community composition at the phylum level in shrimp intestinal tract samples (Figure 3B). The top 10 phyla in terms of relative abundance accounted for 99.77% to 99.90% of the total. These phyla included Proteobacteria, Actinobacteriota, Firmicutes, Verrucomicrobiota, Bacteroidota, Patescibacteria, Planctomycetota, Bdellovibrionota, Cyanobacteria, and Campilobacterota. Among these, Proteobacteria, Actinobacteriota, Firmicutes, and Verrucomicrobiota were the dominant phyla, comprising more than 98% of the community. Compared to the S_CK group, the S_PB group exhibited an increase in the relative abundance of Proteobacteria, Firmicutes, and Verrucomicrobiota, while Actinobacteriota decreased. This indicated that *B. licheniformis* altered the composition of the bacterial community in the shrimp intestinal tracts.

#### 3.4.3 Bacterial community composition at the genus level in the aquaculture water

Analyzing the bacterial community composition at the genus level in the water samples (Figure 3C). The dominant genera, *Aurantimicrobium, Gemmobacter, Paracoccus, Bacillus, Donghicola*, and *Acinetobacter*, had relative abundances ranging from 66.49% to 76.41%. Compared to the W_CK group, the W_PB group showed increased relative abundances of *Gemmobacter, Paracoccus, Bacillus*, and *Donghicola*, while *Aurantimicrobium* and *Acinetobacter* decreased. This indicated that *B. licheniformis* altered the relative abundances of the dominant bacterial genera in the aquaculture water, changing the structure of the bacterial community within the aquaculture environment.

#### 3.4.4 Bacterial community composition at the genus level in shrimp intestinal tracts

Analyzing the bacterial community composition at the genus level in shrimp intestinal tract samples (Figure 3D). The dominant genera, *Gemmobacter, Aurantimicrobium, Fluviicola, Porphyrobacter, Acinetobacter, Flavobacterium, Devosia*, and *Pedomicrobium*, accounted for 77.13% to 84.72% of the total, indicating their predominance in the shrimp intestinal tracts. Compared to the S_CK group, the S_PB group exhibited an increase in the relative abundance of *Aurantimicrobium, Acinetobacter, Devosia*, and *Pedomicrobium*, while *Gemmobacter, Fluviicola, Porphyrobacter*, and *Flavobacterium* decreased. This indicated that *B. licheniformis* also altered the relative abundances of the dominant bacterial genera in the shrimp intestinal tracts, changing the bacterial community composition at the genus level.

#### 3.4.5 Effects of *B. licheniformis* on the distribution of bacterial genera in the aquaculture water and the shrimp intestinal tracts

**Figure 4:**
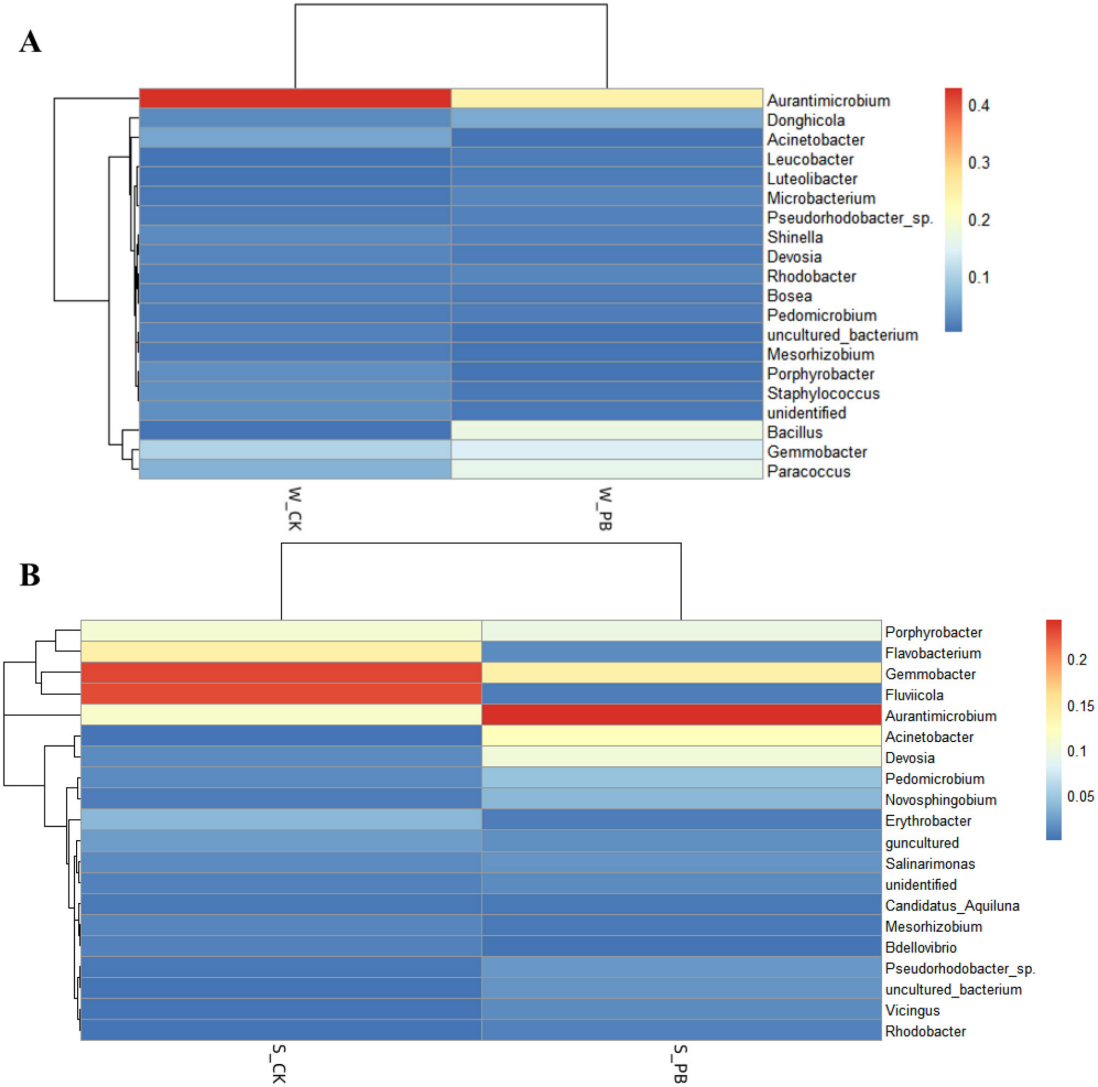
**(A)** Heatmap of different treatment groups in aquaculture water. W_CK: the untreated group of water samples; W_PB: the probiotic treated group of water samples. **(B)** Heatmap of different treatment groups in shrimp intestinal tract. S_CK: the untreated group of shrimp intestinal tract samples; S_PB: the probiotic treated group of shrimp intestinal tract samples.

The top 20 genera were plotted as heatmaps using the R language in the culture water and shrimp intestinal tract samples (Figure 4). Heatmap analysis can further explore the variability of the dominant genera under different treatment conditions in the aquaculture water and shrimp intestinal tracts. Darker colors indicated greater relative abundance of the genus. *Aurantimicrobium* was the absolute dominant genus in both W_CK and W_PB groups. *Gemmobacter* and *Fluviicola* were dominant in the S_CK group, while *Aurantimicrobium* was dominant in the S_PB group. It was obvious that in the aquaculture water, both the W_CK and W_PB groups shared the same dominant bacterial genera, though their relative abundances differed, with the W_CK group exhibiting higher relative abundance. In the shrimp intestinal tracts, the dominant genera differed between the S_CK and S_PB groups, with variations in relative abundance. This indicated that *B. licheniformis* altered the microbial living environment in both the aquaculture water and the shrimp intestinal tracts, subsequently changing their bacterial composition.

#### 3.4.6 Impacts of *B. licheniformis* on the principal components of the bacterial communities in the aquaculture water and the shrimp intestinal tracts

**Figure 5:**
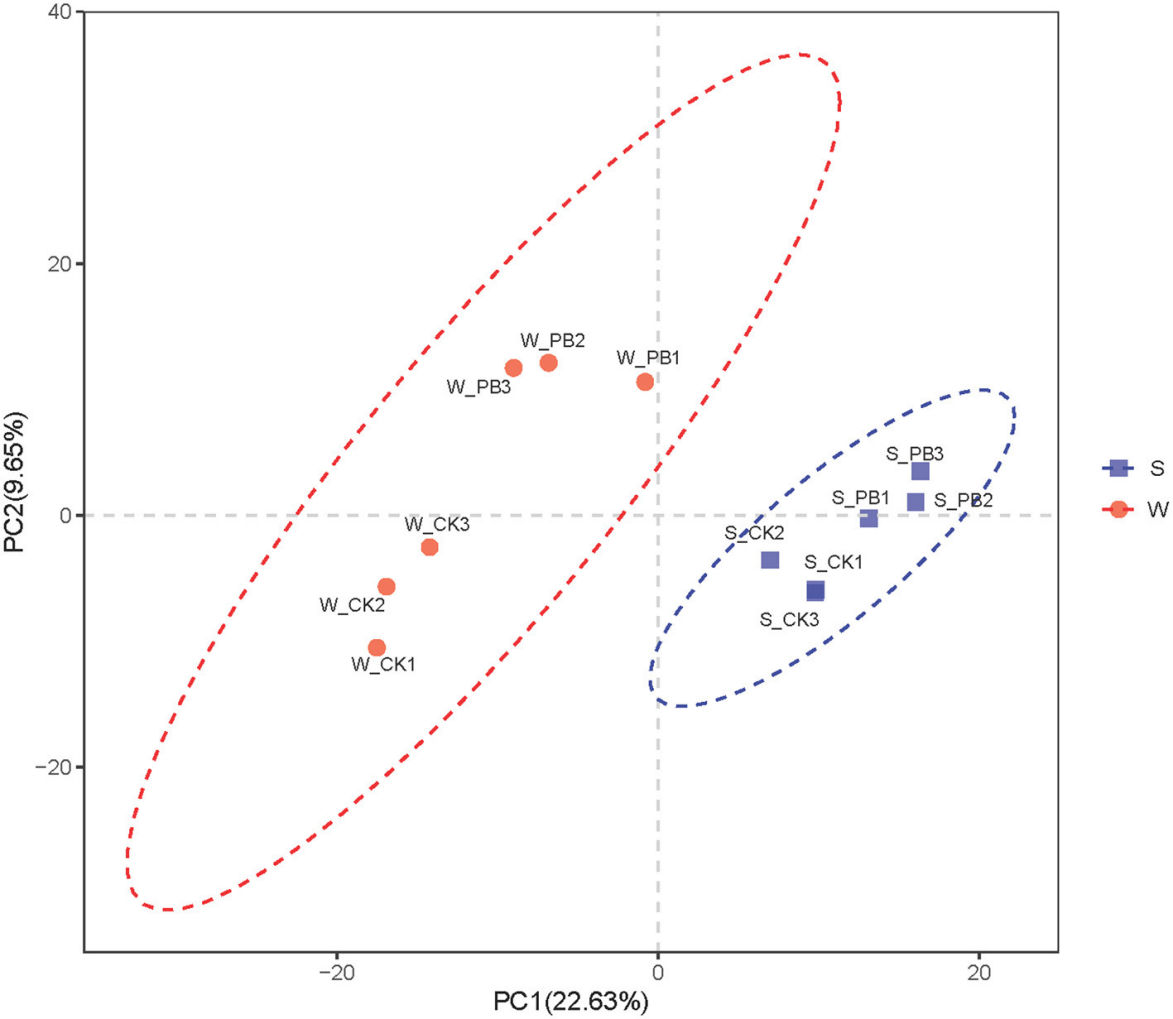
PCA of different treatment groups. The red section represents the water samples, while the blue section represents the shrimp intestinal tract samples. Each point in the graph corresponds to replicates of different treatment groups, and the greater the distance between points, the more significant the difference in bacterial communities. The numbers 1, 2, and 3 represent the three replicate samples. W, water; S, shrimp. Where W_CK; S_CK represents samples obtained from water and shrimp intestine samples obtained from untreated group and W_PB; S_PB probiotic treated group.

PCA was conducted on the aquaculture water and the shrimp intestinal tract samples based on OTUs (Figure 5), effectively illustrating differences in bacterial communities following the application of *B. licheniformis*. In the aquaculture water samples, the inter-parallel distances between the W_CK1, W_CK2, W_CK3 groups, as well as between the W_PB1, W_PB2, W_PB3 groups are close, indicating similarity in bacterial communities among each parallel groups. However, the distance between the W_CK and W_PB groups is considerable, suggesting significant differences between their bacterial communities. Similarly, in the shrimp intestinal tract samples, the inter-parallel distances between the S_CK1, S_CK2, S_CK3 groups, as well as between the S_PB1, S_PB2, S_PB3 groups are close, indicating similarity in bacterial communities among each parallel groups. However, the distance between the S_CK and S_PB groups is considerable, suggesting significant differences between their bacterial communities. In summary, *B. licheniformis* altered the bacterial community structure in both the aquaculture water and the shrimp intestinal tracts.

## 4 Discussion

### 4.1 *B. licheniformis* improved the water quality and promoted the shrimp growth

In the present study, water pH gradually decreased in both the CK and PB groups. Initially, there was no significant difference between the two groups. However, in the later stages, the pH in the PB group was significantly lower than that in the CK group (*P* < 0.05). A reduction in pH over time can be attributed to microbial processes such as organic matter degradation, nitrification, and the resultant increase in carbonic acid (Ebeling et al., 2006). Nimrat et al. (2012) found that the addition of *Bacillus* probiotics to *Penaeus vannamei* resulted in a significant reduction in pH. Hassan et al. (2022) observed a significant decrease in the pH of the ponds after probiotics treatment (*P* < 0.05) compared to pre-treatment levels. These reports were consistent with the results of this study.

In the later stages of aquaculture, the concentrations of ammonia and nitrite in the water of both the CK and PB groups increased considerably. This was attributed to the accumulation of shrimp excreta and uneaten feed in the culture tanks. The proteins and amino acids in these substances contributed to the elevated levels of organic nitrogen in the water, thereby stimulating the production of microbial metabolic byproducts, including ${\text{NO}}_{2}^{-}$-N, ${\text{NO}}_{3}^{-}$-N, and ${\text{NH}}_{4}^{+}$-N (Jackson et al., 2003; Zhou and Boyd, 2015). However, the concentrations of ammonia and nitrite in the PB group were significantly lower than those in the CK group (*P* < 0.05). Microorganisms play diverse ecological roles as producers, consumers, and decomposers, and participate in various nitrogen cycling processes, such as nitrogen fixation (Yousuf et al., 2017), nitrification (Rout et al., 2017), ammonification (Hui et al., 2019), and denitrification (Verbaendert et al., 2011), etc. The microbial preparations used in this experiment were *Bacillus* strains. Monier et al. (2023) found that the application of *Bacillus* species probiotics (*B. subtilis* and *B. licheniformis*) to shrimp aquaculture lowered total ammonia nitrogen (TAN) and NH_3_ in the pond water; Nimrat et al. (2020) found that the addition of *Bacillus* probiotics in the treatment group was associated with remarkable reductions in ammonia-nitrogen and nitrite-nitrogen concentrations in the water than the control group; Wang et al. (2022) similarly found that adding *Bacillus* to the *P. vannamei* culture process significantly reduced ammonia and nitrite contents in the water; These findings illustrated that microecological preparations dominated by *Bacillus* bacteria played a significant role in reducing ammonia and nitrite in water, consistent with this study's results.

This study indicated that compared to the CK group, *B. licheniformis* significantly improved shrimp growth indicators, including length, weight, survival rate, and yield (*P* < 0.05). Furthermore, the feed conversion ratio was significantly reduced (*P* < 0.05). *B. licheniformis* can promote intestinal health in shrimp, enhance the immune system, directly secrete digestive enzymes, and increase the activity of shrimp digestive enzymes, thereby improving feed utilization and promoting shrimp growth and survival. This has been confirmed in many studies and practices. For instance, Mirbakhsh et al. (2021) found that *Bacillus subtilis* administration improved growth and enhanced the immune response of *Penaeus vannamei* during the early hatchery period, thus increasing resistance to bacterial pathogens. Ranjit Kumar et al. (2013) found that dietary supplementation with *Bacillus licheniformis* inhibited pathogenic bacteria in the gut of *Macrobrachium rosenbergii*, enhanced growth and feed efficiency, and improved immune response, thereby increasing protection against bacterial infections. Ziaei-Nejad et al. (2006) also concluded that the use of *Bacillus* at different growth stages of shrimp promoted digestive enzyme activity, and enhanced survival rates and yield. These findings support the idea that *B. licheniformis* promotes shrimp growth and improves survival rates.

### 4.2 *B. licheniformis* increased the diversity of the microbial community in the shrimp aquaculture system, and promoted shrimp growth

The results indicated that *B. licheniformis* increased the number of OTUs, and the Chao1, Shannon, and Simpson indices of the microbial communities in both the aquaculture water and shrimp intestinal tracts, compared to the CK group. This suggested that *B. licheniformis* enhanced bacterial diversity within shrimp farming systems. As a probiotic, *B. licheniformis*, has the ability to improve the aquaculture water quality, promote nutrient cycling and other roles, thus increasing bacterial diversity. The diversity of microbial communities plays a crucial role in maintaining ecological functions, low diversity may indicate poor functional stability of microbial communities, increasing the risk of disease in organisms. The diversity indices of aquaculture water are positively correlated with shrimp health, and variations in intestinal bacterial structure and diversity distinguish between healthy and diseased shrimp. Numerous studies confirmed this: Wu et al. (2016) analyzed the intestinal bacterial community of healthy and diseased shrimp by high-throughput sequencing, the results showed that the bacterial diversity was significantly lower in diseased shrimps than in healthy ones. Yao et al. (2018) and Dai et al. (2018) found that the bacterial Shannon index in the intestinal tract of diseased shrimp was significantly lower than that of healthy shrimp. DU Shi-cong et al. (2019) found that planktonic bacteria in *Penaeus vannamei* culture ponds before the disease emergence exhibited a significantly lower Shannon index than healthy ponds and suggested that a decrease in Shannon index could be a sign of divergence in shrimp health. Reyes et al. (2022) compared the microbiome of *Penaeus vannamei* with high and low survival in shrimp hatchery tanks, the Shannon diversity index was significantly lower at the low-survival tanks.

*B. licheniformis* altered the microbial diversity, bacterial abundance, and community structure in the aquaculture water and the shrimp intestinal tracts, however, the impacts differed between the two environments. In the present study, *B. licheniformis* resulted in changes in the OTUs of bacteria in the aquaculture water and the shrimp intestinal tracts. Notably, in the shrimp intestinal tract samples, the S_PB group shared more OTUs with the S_CK group, indicating a lesser impact of *B. licheniformis* on the shrimp intestinal tract. Compared to the aquaculture water, the microbial community in the shrimp intestines was more stable. Luo et al. (2006) demonstrated that external environmental factors could influence the microbial community structure in the shrimp intestinal tract. However, as shrimp cultivation progressed to a certain stage, the intestinal bacterial community became relatively stable and mature, becoming less susceptible to environmental influences. This finding aligned with the results of this study.

### 4.3 *B. licheniformis* altered bacterial community structure in the culture water and the shrimp intestinal tracts

*B. licheniformis* increased the relative abundance of beneficial bacterial genera. It can enhance synergistic interactions among microorganisms, such as stimulating the growth of other probiotics through the production of bioactive substances, or by establishing symbiotic relationships to participate together in certain biochemical processes. In the culture water samples, it was found that after the addition of *B. licheniformis*, the relative abundance of the genera *Bacillus, Gemmobacter*, and *Paracoccus* was higher than in the CK group. *Bacillus* can inhibit the growth of pathogenic bacteria, as a biological barrier to compete with pathogenic bacteria for nutrients (Tran et al., 2023); The genera *Gemmobacter* (Liu et al., 2021) and *Paracoccus* (Si et al., 2020) have strong denitrification capabilities and effectively decompose organic matter in water, belonging to the beneficial bacteria in shrimp aquaculture process.

*B. licheniformis* reduced the relative abundance of potential pathogens. As a probiotic, *B. licheniformis* competes with potential pathogens for nutrients and living space, and it can produce a broad spectrum of antimicrobial substances. The present study found that *B. licheniformis*, decreased the relative abundance of *Flavobacterium*, a potential pathogen, in shrimp intestinal tracts (Dai et al., 2018). It was observed that *Vibrio* counts in the culture water significantly decreased following the addition of *B. licheniformis* (*P* < 0.05), during the cultivation period between day 10 and day 30. *Vibrio*, a prevalent pathogen in aquatic animal farming environments, is a primary pathogen during the shrimp larval stage and can lead to shrimp mortality (Zhou et al., 2012). The increase of pathogenic bacteria can lead to the occurrence of disease, which has been confirmed in many studies: Rahardjo et al. (2023) investigated microbial composition in rearing media of *Penaeus vannamei* infected by the white feces disease (WFD) and healthy *Penaeus vannamei*. They found that *Vibrio vulnificus* dominated in WFD-infected shrimp pond water. Oxley et al. (2002), Abraham et al. (2004) and Phung et al. (2020) found introducing *Bacillus* into shrimp cultivation significantly reduced the number of *Vibrio*. These findings were consistent with the idea that *B. licheniformis* could increase the relative abundance of beneficial bacterial genera and decrease the relative abundance of potential pathogens.

Some studies have identified the genera such as *Rhodobacter, Pseudomonas*, and *Paracoccus* are the dominant bacterial groups in aquaculture water (Wu, 2016). In the present study, these genera were detectable in the aquaculture water, but at relatively low percentages. The genus *Aurantimicrobium* was found to be the most predominant in the aquaculture water, accounting for the largest proportion of the bacterial community. Numerous studies have found that Gammaproteobacteria is the absolutely dominant bacterial phylum in shrimp intestines, with *Vibrio* and *Aeromonas* being the absolutely dominant genera (Beardsley et al., 2011; Liu et al., 2010). However, in this study, the dominant bacterial genera in shrimp intestines were *Gemmobacter, Fluviicola*, and *Aurantimicrobium*. It is hypothesized that the aforementioned changes might be related to the fact that the present study was conducted in a small-scale farming setting. Microorganisms respond sensitively to environmental changes, and shifts in microbial genera are influenced by variations in the environment, with specific genera changing across different aquaculture environments (Quero et al., 2020). For example, Al-Masqari et al. (2022) cultured *Penaeus vannamei* under different temperature conditions and found that temperature changes significantly influenced the composition of bacterial communities. Furthermore, variations in probiotic species, strains, and application dosages can lead to differences in microbial community composition.

## 5 Conclusion

The results of this study demonstrated that *B. licheniformis* could improve the water quality in *P. vannamei* aquaculture, enhance shrimp growth performance and disease resistance, and regulate the microbial communities in both the cultivation water and the shrimp intestinal tract. Specifically, the abundance of potentially harmful bacteria was reduced, while the abundance of beneficial bacteria increased. These findings indicated that *B. licheniformis* has great potential for practical application in the large-scale cultivation of *P. vannamei*.

## Data Availability

The data presented in the study are deposited in the NCBI Short Read Archive (SRA), accession numbers SRX28901524-SRX28901535.
